# Effects of Antibiotic Stewardship Program on Antibiotic Consumption and the Incidence of *Clostridioides difficile* Infection

**DOI:** 10.3390/antibiotics15020112

**Published:** 2026-01-23

**Authors:** Joung Ha Park, Juhee Kim, Juyeon Lee, Hyemin Chung, Min-Chul Kim

**Affiliations:** 1 Division of Infectious Diseases, Department of Internal Medicine, Chung-Ang University Gwangmyeong Hospital, 110 Deokan-ro, Gwangmyeong-si 14353, Republic of Korea; pjha89@cauhs.or.kr (J.H.P.); hyeminchung@cauhs.or.kr (H.C.); 2 Department of Pharmacy, Chung-Ang University Gwangmyeong Hospital, 110 Deokan-ro, Gwangmyeong-si 14353, Republic of Korea; 20085@cauhs.or.kr (J.K.); 20876@cauhs.or.kr (J.L.)

**Keywords:** antibiotics, days of therapy, antibiotics stewardship program, *Clostridioides difficile* infection

## Abstract

**Background/Objectives**: Growing concerns about antibiotic-associated adverse events, including *Clostridioides difficile* infection, prompted implementation of an antibiotic stewardship program (ASP) in South Korea in November 2024. One year post-implementation, we evaluated changes in antibiotic consumption and *C. difficile* infection incidence. **Methods**: This study was conducted at Chung-Ang University Gwangmyeong Hospital, South Korea. Segmented regression and interrupted time series analyses were performed using weekly data on antibiotic use (days of therapy [DOT] per 1000 patient-days) and *C. difficile* infection or colonization (cases per 1000 patient-days) over 157 weeks (November 2022–October 2025). Weeks 1–105 defined the pre-ASP period, and weeks 106–157 the post-ASP period. A 4-week lag between antibiotic use and subsequent *C. difficile* infection was hypothesized. **Results**: Before ASP, weekly total antibiotic use increased (*β*_1_ = 1.14, 95% CI, 0.76 to 1.51, *p* < 0.001). After ASP, the slope decreased significantly (*β*_3_ = −1.50, 95% CI −2.62 to −0.39, *p* = 0.009), consistent across anti-pseudomonal penicillins and cephalosporins and fluoroquinolones. Pre-ASP *C. difficile* incidence increased (α_1_ = 0.01, 95% CI, 0.01 to 0.02, *p* < 0.001); the upward trend attenuated post-ASP, though slope change was not significant (α_3_ = −0.01, 95% CI, −0.03 to 0.004, *p* = 0.13). An increase of 1 DOT per 1000 patient-days was associated with a 0.005-case increase in *C. difficile* infection incidence after 4 weeks. **Conclusions**: The observed effects of proactive ASP strategies underscore the importance of maintaining stewardship in clinical practice. Further studies are warranted to assess the sustainability of these findings and evaluate additional factors influencing *C. difficile* infection incidence.

## 1. Introduction

*Clostridioides difficile* infection is a major cause of healthcare-associated diarrhea and can range from mild disease to life-threatening complications. According to recent data from the Health Insurance Review and Assessment Service in South Korea, the incidence of *C. difficile* infection increased from 1.0 to 5.1 per 10,000 patient-days in tertiary hospitals and from 0.6 to 5.0 per 10,000 patient-days in general hospitals over a 13-year period (from 2008 to 2020), with similar global trends reported [1,2,3]. As *C. difficile* infection can be transmitted to other patients through both direct and indirect contact, implementing infection control strategies, including contact precautions and hand hygiene, is essential for preventing disease transmission in healthcare settings. In addition, antibiotic consumption is a well-established risk factor for *C. difficile* infection because antibiotics can alter the gut microbiota, which mediates colonization resistance against *C. difficile* [4,5]. Therefore, minimizing unnecessary antibiotic use is a key strategy for preventing *C. difficile* infections.

Growing concerns about the adverse effects of antibiotics, such as *C. difficile* infection and the emergence of multidrug-resistant organisms (MDRO), have prompted the worldwide adoption of antimicrobial stewardship programs (ASP). Since 2014, the US Centers for Disease Control and Prevention (CDC) has released core elements of hospital antibiotic stewardship programs to optimize antibiotic use [6]. In general, strategies to improve antibiotic prescribing are categorized into restriction (pre-prescription approval) and enablement interventions (education, post-prescription feedback, and audits) [7,8]. At our center, which opened in March 2022, we controlled the use of broad-spectrum antibiotics, such as carbapenems, through a policy requiring preauthorization for their prescription. To reflect global trends, South Korea launched a nationwide ASP pilot program in November 2024 to reinforce antibiotic stewardship. One year after ASP implementation in our center, we aimed to evaluate changes in both antibiotic consumption and *C. difficile* infection incidence before and after ASP introduction. Furthermore, using an interrupted time-series analysis of real-world data, we assessed the impact of changes in antibiotic use on the incidence of *C. difficile* infection.

## 2. Results

### 2.1. Effect of ASP on Antibiotic Consumption

**Figure 1:**
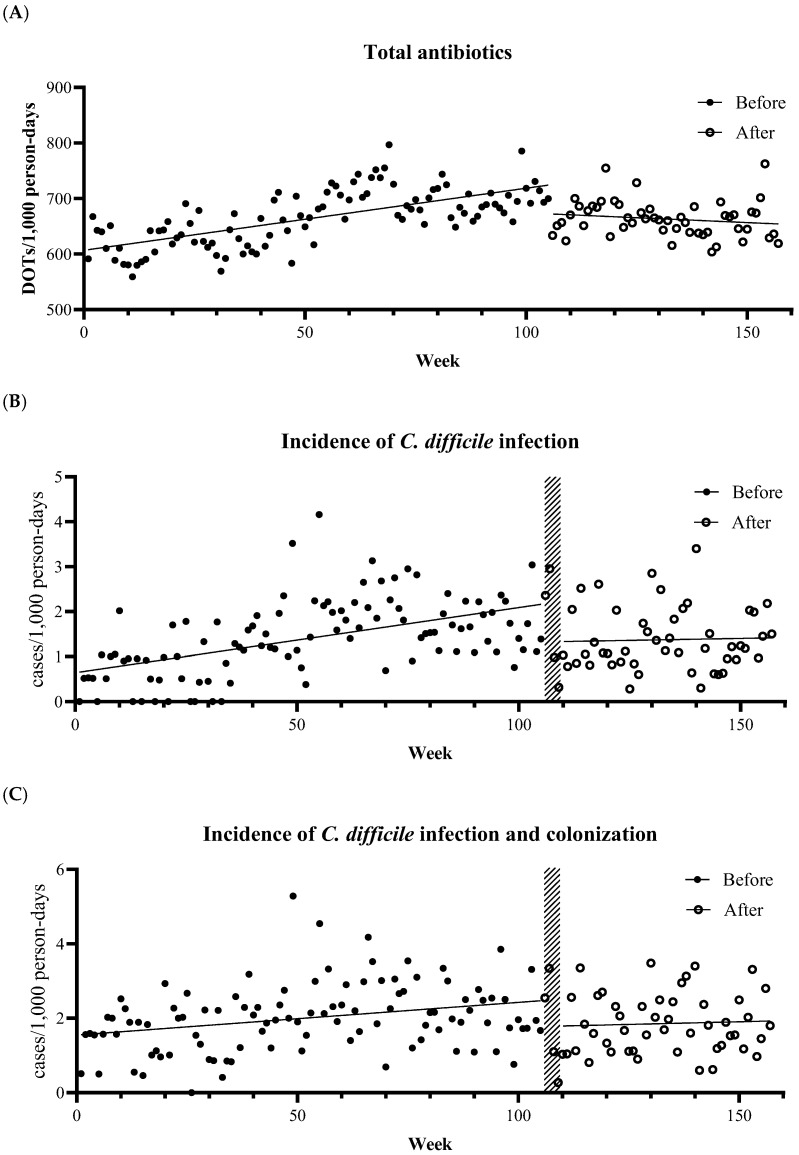
Weekly trends in total antibiotic use and CDI incidence with segmented lines around ASP implementation.

Over 157 weeks, the total antibiotic use (days of therapy [DOT] per 1000 patient-days) showed a segmented trend around ASP initiation (week 106) (Table S1 and Figure 1A). Before ASP, weekly total antibiotic use increased (*β*_1_ = 1.14, 95% CI 0.76 to 1.51, *p* < 0.001). After ASP implementation, the change in slope was negative (*β*_3_ = −1.50, 95% CI −3.62 to −0.39, *p* = 0.009). In segmented regression, the post-ASP slope equals the sum of the pre-ASP slope (*β*_1_) and the slope change after ASP (*β*_3_). Based on the above estimates, the post-ASP slope is *β*_1_ + *β*_3_ = −0.36 DOT per 1000 patient-days. The class-specific consumption results were consistent for penicillins, anti-pseudomonal penicillins and cephalosporins, and fluoroquinolones (Table S2 and Figure S1). In contrast, cephalosporin use did not change significantly during the pre-ASP period but increased in the post-ASP period. The use of carbapenems and glycopeptides did not change significantly in either the pre- or post-ASP periods.

### 2.2. Incidence of C. difficile Infection and Colonization

Patients with *C. difficile* infection or colonization had a median age of 69 years (interquartile range, 53–81). To account for a biologically plausible delay after ASP implementation, weeks 106–109 were excluded as wash-in periods. These observations were plotted as points but were excluded from trend fitting (Figure 1B,C), and the post-ASP segment was fitted from week 110 onward. While pre-ASP *C. difficile* infection incidence increased (α_1_ = 0.01, 95% CI 0.01 to 0.02, *p* < 0.001), the change in slope after ASP was not significant (α_3_ = −0.01, 95% CI −0.03 to 0.004, *p* = 0.13) (Table S3A and Figure 1B). The trends in *C. difficile* infection and colonization incidence were similar (Table S3B and Figure 1C).

### 2.3. Lagged Effect of Antibiotic Use on C. difficile Infection

**Figure 2:**
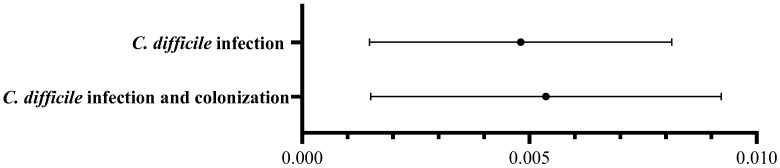
Effect of total antibiotic use on the incidence of *C. difficile* infection and colonization at a 4-week lag–forest plot of GLS coefficients (β, 95% confidence interval).

After additional analysis of the total effect of antibiotic use on *C. difficile* infection occurrence with 1-, 2-, 4-, and 8-week lags, we selected a 4-week lag for the primary analysis. In the primary ITS exposure–response model with a 4-week lag for antibiotic use, we analyzed the relationship between antibiotic use (DOT per 1000 patient-days) 4 weeks earlier and subsequent *C. difficile* infection and colonization after adjusting for calendar time. *C. difficile* infection incidence increased by 0.005 cases per 1000 patient-days with an increase of 1 DOT per 1000 patient-days of total antibiotics 4 weeks earlier. *C. difficile* infection and colonization incidence also increased by 0.005 cases per 1000 patient-days with an increase of 1 DOT per 1000 patient-days in total antibiotics (Figure 2).

### 2.4. Sensitivity Analysis—Antibiotic Class-Specific Effects on C. difficile Infection Occurrence at Fixed Total Antibiotic Use

We performed a sensitivity analysis to estimate the compositional effect, in which an increase of 1 DOT in a given antibiotic class was interpreted as displacing 1 DOT from the remaining classes while holding the total DOT constant. When total antibiotic use was held constant, an additional 1 DOT per 1000 patient-days of fluoroquinolone use was associated with 0.02 more cases of *C. difficile* infection and colonization per 1000 patient-days (Figure S2). However, the association between fluoroquinolone use and *C. difficile* infection incidence was not statistically significant. In contrast, an additional 1 DOT per 1000 patient-days of cephalosporin use was associated with 0.008 fewer cases of *C. difficile* infection per 1000 patient-days and 0.01 fewer cases of *C. difficile* infection and colonization per 1000 patient-days. Similarly, glycopeptide use was associated with 0.02 fewer cases of *C. difficile* infection and colonization per 1000 patient-days per 1 DOT increase. The other classes did not show significant associations with *C. difficile* infection or colonization.

## 3. Discussion

### 3.1. Main Findings

In this single-center ITS analysis conducted over 157 consecutive weeks, we identified three main findings. First, total antibiotic consumption decreased following ASP implementation compared to the pre-ASP period, which relied on restriction strategies. In particular, the use of anti-pseudomonal penicillins and cephalosporins, and fluoroquinolones markedly declined after the intervention. Second, although the incidence of *C. difficile* infection steadily increased before ASP implementation, this upward trend was attenuated after the intervention; however, the change in slope did not reach statistical significance. The incidence of *C. difficile* infection and colonization was similar. Third, the exposure–response model demonstrated a lagged association between antibiotic use and *C. difficile* infection occurrence. The incidence of *C. difficile* infection increased by 0.005 cases per 1000 patient-days for every 1 DOT per 1000 patient-days increase in total antibiotic consumption four weeks earlier.

### 3.2. Interpretation

We demonstrated that ASP implementation was associated with decreased total antibiotic consumption, showing an immediate decline at the time of implementation, followed by a significantly decreased slope thereafter. A previous systematic review also reported an approximately 10% decrease in antibiotic prescriptions following ASP implementation, consistent with our findings [9]. The marked immediate decline at the time of implementation was likely attributable to heightened institutional awareness and vigilance among prescribing physicians. We also evaluated antibiotic class-specific changes after ASP implementation. The use of carbapenems and glycopeptides did not change significantly in the post-ASP period. It is likely that broad-spectrum antibiotics, such as carbapenems and glycopeptides, were already controlled by pre-existing restriction strategies. Our center participated in the Korea National Antimicrobial Use Analysis System (KONAS) in November 2024 and reported quarterly carbapenem consumption. According to the KONAS report, carbapenem use was already lower (39.6 DOT per 1000 patient-days) than that of other hospitals of similar size (61.1 DOT per 1000 patient-days), even before ASP implementation. These findings suggest that pre-existing restriction strategies effectively limit the use of targeted antibiotics, including carbapenems. Therefore, further reductions in carbapenem use after ASP implementation may have been inherently limited. Previous studies have shown that restriction strategies markedly reduce targeted antibiotic use [10,11], whereas discontinuation of these strategies could lead to a return to prior patterns of antibiotic consumption [12]. However, these restriction strategies may not affect the use of other antibiotics and may even lead to a balloon effect [13]. Given this limitation, we endorsed reinforced ASP policies in November 2024. Consequently, the use of anti-pseudomonal penicillins and cephalosporins, and fluoroquinolones markedly decreased. The increased use of total cephalosporins may be explained by the substitution effect following de-escalation strategies using narrow-spectrum cephalosporins such as cefazolin or ceftriaxone, which is consistent with the goals of ASP [14]. Unlike previous restriction strategies that relied on prescription request forms for restricted antibiotics, the newly implemented ASP adopted more active approaches by monitoring all antibiotic use daily and providing repeated education and prompt feedback on inappropriate prescriptions to prescribing physicians. This proactive ASP approach appears to be effective in reducing unnecessary antibiotic use.

Antibiotic exposure is a well-known risk factor for *C. difficile* infection, and a time lag between antibiotic use and subsequent *C. difficile* infection is commonly observed [4,15,16]. The time-lag period aligns with the incubation period for *C. difficile* infection and the time required for antibiotic-induced dysbiosis to facilitate spore germination and toxin production in the gut, supporting the biological plausibility [5]. Thus, in this study, *C. difficile* infection incidence was analyzed considering a 4-week lag effect following antibiotic consumption. In this model, *C. difficile* infection incidence increased by 0.005 cases per 1000 patient-days for every 1 DOT per 1000 patient-days increase in antibiotic use. This finding is consistent with those of previous studies showing positive correlations between antibiotic use and *C. difficile* infection incidence [17,18,19]. Previous systematic reviews have identified a 32–52% reduction in *C. difficile* infection after ASP implementation [17,18]. In addition, a cross-sectional analysis demonstrated a 4.4% increase in hospital-onset *C. difficile* infection every 50 DOT per 1000 patient-day increase in total antibiotic use [19]. Although some studies reported that *C. difficile* infection incidence did not decline despite reductions in antibiotic consumption [20,21], the effect size observed in our study—an additional 0.25 cases per 1000 patient-days for every 50 DOT increase per 1000 patient-days—supports the notion that reducing antibiotic consumption through ASP meaningfully contributes to lowering *C. difficile* infection incidence. However, *C. difficile* infection is influenced not only by antibiotic consumption but also by other factors, including diagnostic testing strategies, infection control measures, and patients’ clinical characteristics [22]. As we could not analyze the effects of these confounding factors on *C. difficile* infection, the observed change in incidence cannot be attributed solely to reductions in antibiotic use.

Several previous studies have identified specific antibiotic classes that are more likely to induce *C. difficile* infection, particularly second- and third-generation cephalosporins, fluoroquinolones, and clindamycin [22,23]. In the present study, although the independent effect of each antibiotic class on *C. difficile* infection incidence could not be demonstrated, we assessed the relative effect of class-specific antibiotic use under the assumption that total antibiotic consumption remained constant. In this context, an increase of 1 DOT in a specific class implies a corresponding decrease of 1 DOT in the other classes. Similarly to previous studies, an increase of 1 DOT of fluoroquinolones, a well-known high-risk class [22,24], significantly increased the incidence of *C. difficile* infection and colonization in our analysis. In contrast, the increased use of cephalosporins and glycopeptides was associated with a decrease in *C. difficile* infection incidence. This finding differs from that of a previous study, which classified second- and third-generation cephalosporins as high-risk antibiotics for *C. difficile* infection [24]. These discrepancies may be attributed to the fact that this study was unable to independently evaluate the inherent effects of each antibiotic class.

### 3.3. Limitations of the Study

This study had some limitations. First, the study was performed at a single center. As the hospital is a recently established institution in its fourth year of operation, the generalizability of the findings may be limited. However, studies involving large populations and multiple institutions often introduce substantial heterogeneity and confounding factors. The results of this study nonetheless provide meaningful insights. Second, although *C. difficile* infection can be influenced by various factors, such as proton-pump inhibitor use, length of hospital stay, and underlying diseases, we did not adjust for these potential confounders and focused on antibiotic consumption. Moreover, infection control measures (e.g., hand hygiene compliance and isolation protocols) were not considered, although the hospital’s infection control policy against *C. difficile* infection remained consistent throughout the study period. Further studies are required to evaluate the effects of these variables on *C. difficile* infection using multivariable ITS analyses. Third, we assessed antibiotic exposure solely using DOT and did not evaluate the defined daily dose (DDD). Because DOT does not reflect prescribed doses, we could not examine dose-dependent associations between antibiotic exposure and *C. difficile* infection. Finally, we did not consider the clinical severity of *C. difficile* infection, which may provide additional insights into the association between antibiotic consumption and clinical outcomes. In addition, as strain-specific virulence (e.g., ribotype 027) and antibiotic resistance data were available, we were unable to evaluate their contribution to the observed association between antibiotic consumption and *C. difficile* infection and colonization.

### 3.4. Conclusions

In conclusion, proactive ASP strategies were effective in significantly decreasing overall antibiotic consumption. Notably, the use of anti-pseudomonal penicillins and cephalosporins, and fluoroquinolones—which were not adequately controlled under previous restriction strategies—showed a substantial decline. Furthermore, this reduction in antibiotic consumption led to a decrease in the incidence of *C. difficile* infection or colonization. These findings highlight the importance of proactive ASP strategies in clinical practice. Further studies with longer follow-up periods are warranted to assess the sustainability of these results and evaluate additional factors that may influence *C. difficile* infection occurrence.

## 4. Materials and Methods

### 4.1. Study Design

This study was conducted at Chung-Ang University Gwangmyeong Hospital in Gyeonggi-do, Republic of Korea. Our center opened in March 2022. Since then, we have limited the prescription of broad-spectrum antibiotics, including ceftazidime-avibactam, ceftolozane-tazobactam, carbapenems, glycopeptides, colistin, daptomycin, linezolid, and tigecycline, to 3 days without approval from infectious disease specialists. In addition, clinicians discuss infectious disease cases in formal consultations with infectious disease specialists. After implementing the nationwide ASP pilot program in November 2024, we adopted a reinforced ASP policy, as described in Table 1. We performed segmented regression and interrupted time-series (ITS) analyses using weekly data on antibiotic use and cases of *C. difficile* infection and colonization over 157 consecutive weeks from November 2022 to October 2025 (weeks 1–157). We defined the pre-ASP period as weeks 1–105, and the post-ASP period as weeks 106–157. This study was approved by the Institutional Review Board of our center. The requirement for written informed consent was waived due to the retrospective nature of the study.

### 4.2. Definition

All antibiotics were monitored daily and are listed in Table S4. We included all antibiotics used in our hospital except oral vancomycin, as this drug is specifically administered for the treatment of *C. difficile* infections. Fidaxomicin, which is a treatment of choice of *C. difficile* infection, was not included in the analysis because it was not available in South Korea during the study period. We also analyzed the DOT according to antibiotic class, including penicillins, cephalosporins, carbapenems, glycopeptides, and fluoroquinolones. The anti-pseudomonal penicillins and cephalosporins included piperacillin-tazobactam, ceftazidime, and cefepime. DOT was defined as the number of days for which a patient received any specific antibiotic. Any amount of agent administered on a given day was counted as one DOT. *C. difficile* infection was defined as the presence of clinical symptoms (loose stool or diarrhea) and positive results for *C. difficile* toxin polymerase chain reaction (PCR) or enzyme immunoassay (EIA). *C. difficile* colonization was defined as cases with negative results for *C. difficile* toxin PCR and EIA and a positive result for *C. difficile* culture, since culture at our center identifies both toxigenic and non-toxigenic strains.

### 4.3. Outcomes

We described the weekly use of total antibiotics, quantified as DOT per 1000 patient-days, during the pre- and post-ASP periods. We also examined the antibiotic class-specific DOT per 1000 patient-days, including penicillins, cephalosporins, anti-pseudomonal penicillins and cephalosporins, carbapenems, fluoroquinolones, and glycopeptides. We analyzed the weekly incidence of *C. difficile* infection and colonization, described as cases per 1000 patient-days. We excluded weeks 106–109 from the analysis of the weekly incidence of *C. difficile* infection and colonization because this period is considered a wash-in period. Furthermore, we analyzed the impact of changes in DOT per 1000 patient-days on changes in *C. difficile* infection and colonization with a 4-week lag.

### 4.4. Statistical Analysis

We performed a segmented regression analysis of the ITS to assess weekly observations over 157 consecutive weeks, aiming to evaluate changes associated with ASP implementation and quantify the lagged association between antibiotic use and *C. difficile* infection and colonization [25,26]. Week 106 was the first post-ASP week (the ASP was implemented immediately after week 105).

#### 4.4.1. Time Variables

Let *t* = 1, …, 157 index weeks and define:

- time*_t_* = *t*.
- Post-ASP from week 106: interv106*_t_* = 1 (*t* ≥ 106) and post106*_t_* = max (0,  *t* − 105).
- For wash-in handling when modeling *C. difficile* infection and colonization trends from week 110: interv110*_t_* = 1 (*t* ≥ 110) and post110*_t_* = max (0, *t* − 109).
- Lagged exposure for the exposure–response models: DOT*_t_*_−*4*_ (4-week lag).

#### 4.4.2. Effect of ASP on Antibiotic Consumption

To estimate the changes in the level and slope of antibiotic consumption following ASP implementation, we fit the model as follows:

DOT*t* = *β*_0_ + *β*_1_ time*_t_* + *β*_2_ interv106*_t_* + *β*3 post106*_t_* + *ε_t_*,

where *β*_1_ denotes the pre-ASP slope, *β*_2_ the immediate level change at week 106, and *β*_3_ the change in slope after ASP (post-ASP slope = *β*_1_ + *β*_3_). The same specifications were applied to the class-specific DOT for descriptive inference.

#### 4.4.3. Incidence of *C. difficile* Infection and Colonization

To describe ASP-associated changes in *C. difficile* infection, we applied a 4-week wash-in immediately after ASP initiation and excluded outcome weeks 106–109. We considered that this period might have been affected by the DOT during the pre-ASP period. Thus, *C. difficile* infection trends were modeled from week 110 onward using the following equation:

CDI*t* = *α*_0_ + *α*_1_ time*_t_* + *α*_2_ interv110*_t_* + *α*_3_ post110*_t_* + *ε_t_*,

Fit on weeks *t* ≤ 105 or *t* ≥ 110. The incidence of *C. difficile* colonization was modeled using the same specifications.

#### 4.4.4. Lagged Effect of Antibiotic Use on *C. difficile* Infection and Colonization

We hypothesized that antibiotic use could affect subsequent *C. difficile* infection with a 4-week lag [15,16]. To select an optimal single-lag exposure–response structure, ITS models incorporating lag periods of 1, 2, 4, and 8 weeks were compared using maximum likelihood estimation and the Akaike and Bayesian information criteria (AIC/BIC). The lag minimizing both criteria was selected and refitted using the restricted maximum likelihood (REML) for inference. To quantify the relationship between antibiotic use and subsequent *C. difficile* infections, we modeled the following:

CDI*_t_* = *γ*_0_ + *γ*_1_ time*_t_* + *γ*2 interv106*_t_* + *γ*_3_ post106*_t_* + *γ*_4_ DOT*_t_*_−4_ + *ε_t_*,

Using all weeks with defined lagged exposure (i.e., *t* ≥ 5). The coefficient γ4 represents the absolute change in *C. difficile* infection incidence (cases per 1000 patient-days) associated with a 1-unit increase in total antibiotic use (DOT per 1000 patient-days) 4 weeks earlier, adjusted for underlying time trends and ASP phase. Changes in *C. difficile* colonization incidence were estimated using the same specifications.

#### 4.4.5. Sensitivity Analysis: Antibiotic Class-Specific Effects on *C. difficile* Infection Occurrence at Fixed Total DOT

To evaluate the effect of antibiotic class-specific composition on subsequent *C. difficile* infections, we performed class-composition ITS modeling. The total DOT and one class-specific DOT, each lagged by 4 weeks, were entered concurrently in each model. For each antibiotic class *k*, the model was as follows:

CDI*_t_* = *δ*_0_ + *δ*_1_ time*_t_* + *δ*_2_ interv106*_t_* + *δ*_3_ post106*_t_* + *δ*_4_ totalDOT*_t_*_−*4*_ + *θ_k_* classDOT*_k_*_,*t*−*4*_ + *ε_t_*,

where *θ_k_* estimates the absolute change in *C. difficile* infection incidence (cases per 1000 patient-days) for a 1 DOT per 1000 patient-days increase in class *k*, holding total DOT constant. This represents a compositional effect, as the increased use of one class substitutes for others. The same approach was applied to *C. difficile* colonization incidence.

#### 4.4.6. Error Structure and Estimation

All ITS models were estimated by generalized least squares (GLS) with first-order autoregressive errors to account for serial correlation in weekly residuals: *ε_t_* = *ϕε_t_*_−*1*_ + *u_t_* with *u_t_*~*N*(0, σ^2^). The models were fitted using REML. Regression coefficients were reported with two-sided *p*-values and 95% confidence intervals (CIs), and the AR(1) parameter *ϕ* was presented to quantify week-to-week residual correlation. Model adequacy was evaluated by inspecting normalized residuals (ACF/PACF) and applying the Durbin–Watson and Ljung–Box tests for residual autocorrelation. Models were excluded if the diagnostic results were unsatisfactory.

#### 4.4.7. Software

Analyses were conducted using R software (version 4.5.1). Descriptive segmented lines were plotted using GraphPad Prism software (version 10.5.0). Plots were used for visualization only, whereas statistical inference was derived from the GLS ITS models.

## Figures and Tables

**Table 1:** Antimicrobial stewardship program (ASP) policy in our center.

Pre-ASP period (week 1–105)
Consultation with infectious disease specialists Implementation of restriction and approval systems for broad-spectrum (carbapenems, glycopeptides, linezolid, aminoglycosides, and colistin) and later-generation antimicrobial administration (ceftazidime/avibactam and ceftolozane/tazobactam)
Post-ASP (week 106–157)
Standard policy Consultation with infectious disease specialists Reinforcement of restriction and approval systems for broad-spectrum and later-generation antimicrobial administration Augmented policy Establishment of a multidisciplinary antimicrobial stewardship team that includes infectious disease specialists and pharmacists Development of institutional clinical guidelines for infectious diseases and renal dose adjustment of antibiotics Ongoing education and training for healthcare workers—including prescribers, pharmacists, and nurses—on antimicrobial stewardship, antimicrobial resistance, and optimal antimicrobial use Review and monitoring of antimicrobial prescriptions (daily monitoring of antibiotic days of therapy [DOT]) and direct feedback to prescribers Implementation of point-of-care interventions: ✔ Dose optimization (i.e., therapeutic drug monitoring of vancomycin and voriconazole) ✔ Use of antimicrobial agents for appropriate durations ✔ Intervention on unnecessary combination therapy involving multiple antimicrobial agents Pathogen-directed antimicrobial optimization following positive blood cultures Monitoring of the incidence of *Clostridioides difficile* infection and colonization

## Data Availability

The datasets generated and analyzed during the current study are not publicly available due to patient confidentiality but are available from the corresponding author on reasonable request.

## Supplementary Materials

The following supporting information can be downloaded at: https://www.mdpi.com/article/10.3390/antibiotics15020112/s1, Figure S1: Weekly trends in the use of antibiotic classes with segmented lines around ASP implementation; Figure S2: Antibiotic class-specific effects on *C. difficile* infection and colonization at fixed total DOT; Table S1: Segmented GLS regression of the weekly use of antibiotic classes (DOT per 1000 patient-days) before and after ASP implementation; Table S2: Antibacterial classification based on ATC code; Table S3: Segmented GLS regression of weekly *Clostridioides difficile* infection and colonization incidence (cases per 1000 patient-days) before and after ASP implementation; Table S4: Antibacterial classification based on ATC code.

## Abbreviations

The following abbreviations are used in this manuscript:

ASP	Antibiotic stewardship program
DOT	Days of therapy
MDRO	Multidrug-resistant organisms
CDC	Centers for Disease Control and Prevention
ITS	Interrupted time-series
PCR	Polymerase chain reaction
EIA	Enzyme immunoassay
AIC/BIC	Akaike and Bayesian information criteria
REML	Restricted maximum likelihood
GLS	Generalized least squares
CIs	Confidence intervals
KONAS	Korea National Antimicrobial Use Analysis System
