# Supplementary material for: Effects of Antibiotic Stewardship Program on Antibiotic Consumption and the Incidence of Clostridioides difficile Infection
Source: Antibiotics (Basel). 2026 Jan 23;15(2):112. doi: 10.3390/antibiotics15020112 (PMC12937197; doi:10.3390/antibiotics15020112)
Supplement: Supplementary file 1 [file antibiotics-15-00112-s001.zip › antibiotics-4082647-supplementary.docx]

**Table S1. Segmented GLS regression of weekly total antibiotic use (DOT per 1,000 patient-days) before and after ASP implementation**

| **Predictor** | ***β*** | **95% CI** | ***p*-value** |
| --- | --- | --- | --- |
| **Intercept (*β*₀)** | 605.95 | 583.05 to 628.84 | <0.001 |
| **time (*β*₁)** | 1.14 | 0.76 to 1.51 | <0.001 |
| **intervention (*β*₂)** | -53.21 | -90.34 to -16.08 | 0.006 |
| **posttime (*β*₃)** | -1.50 | -2.62 to -0.39 | 0.009 |

Segmented GLS regression of weekly total antibiotic use with AR(1) errors; *β*₂ indicates the immediate level change, and *β*₁ and *β*₁+*β*₃ indicate the pre- and post-intervention slopes, respectively.

**Table S2. Segmented GLS regression of the weekly use of antibiotic classes (DOT per 1,000 patient-days) before and after ASP implementation**

**(A) Penicillins**

| **Predictor** | **β** | **95% CI** | ***p*-value** |
| --- | --- | --- | --- |
| **Intercept (β₀)** | 109.75 | 99.41 to 120.09 | <0.001 |
| **time (β₁)** | 0.29 | 0.12 to 0.46 | 0.001 |
| **intervention (β₂)** | 0.004 | -16.52 to 16.53 | >0.99 |
| **posttime (β₃)** | -0.95 | -1.45 to -0.44 | <0.001 |

**(B) Cephalosporins**

| **Predictor** | **β** | **95% CI** | ***p*-value** |
| --- | --- | --- | --- |
| **Intercept (β₀)** | 294.80 | 281.20 to 308.40 | <0.001 |
| **time (β₁)** | -0.09 | -0.32 to 0.13 | 0.40 |
| **intervention (β₂)** | -14.50 | -36.68 to 7.67 | 0.20 |
| **posttime (β₃)** | 0.79 | 0.13 to 1.45 | 0.02 |

**(C) Anti-pseudomonal penicillins and cephalosporins**

| **Predictor** | **β** | **95% CI** | ***p*-value** |
| --- | --- | --- | --- |
| **Intercept (β₀)** | 98.35 | 87.02 to 109.67 | <0.001 |
| **time (β₁)** | 0.23 | 0.05 to 0.41 | 0.01 |
| **intervention (β₂)** | 4.11 | -13.75 to 21.97 | 0.65 |
| **posttime (β₃)** | -1.14 | -1.68 to -0.59 | <0.001 |

**(D) Carbapenems**

| **Predictor** | **β** | **95% CI** | ***p-*value** |
| --- | --- | --- | --- |
| **Intercept (β₀)** | 39.88 | 33.40 to 46.36 | <0.001 |
| **time (β₁)** | 0.03 | -0.07 to 0.14 | 0.55 |
| **intervention (β₂)** | -2.32 | -12.81 to 8.17 | 0.66 |
| **posttime (β₃)** | 0.02 | -0.30 to 0.33 | 0.92 |

**(E) Fluoroquinolones**

| **Predictor** | **β** | **95% CI** | ***p*-value** |
| --- | --- | --- | --- |
| **Intercept (β₀)** | 54.92 | 47.89 to 61.95 | <0.001 |
| **time (β₁)** | 0.29 | 0.18 to 0.41 | <0.001 |
| **intervention (β₂)** | -16.91 | -28.35 to -5.47 | 0.004 |
| **posttime (β₃)** | -0.70 | -1.04 to -0.36 | <0.001 |

**(F) Glycopeptides**

| **Predictor** | **β** | **95% CI** | ***p*-value** |
| --- | --- | --- | --- |
| **Intercept (β₀)** | 34.11 | 29.93 to 38.28 | <0.001 |
| **time (β₁)** | 0.001 | -0.07 to 0.07 | 0.97 |
| **intervention (β₂)** | -2.34 | -9.20 to 4.53 | 0.51 |
| **posttime (β₃)** | 0.03 | -0.23 to 0.18 | 0.80 |

**Table S3. Segmented GLS regression of weekly *Clostridioides difficile* infection and colonization incidence (cases per 1,000 patient-days) before and after ASP implementation**

**(A) Incidence of *C. difficile* infection**

| **Predictor** | ***α*** | **95% CI** | ***p*-value** |
| --- | --- | --- | --- |
| **Intercept (α₀)** | 0.64 | 0.34 to 0.94 | <0.001 |
| **time (α₁)** | 0.01 | 0.01 to 0.02 | <0.001 |
| **intervention (α₂)** | -0.89 | -1.43 to -0.35 | 0.002 |
| **posttime (α₃)** | -0.01 | -0.03 to 0.004 | 0.13 |

**(B) Incidence of *C. difficile* infection and colonization**

| **Predictor** | ***α*** | **95% CI** | ***p*-value** |
| --- | --- | --- | --- |
| **Intercept (α₀)** | 1.55 | 1.21 to 1.88 | <0.001 |
| **time (α₁)** | 0.009 | 0.003 to 0.01 | 0.002 |
| **intervention (α₂)** | -0.79 | -1.33 to -0.11 | 0.02 |
| **posttime (α₃)** | 0.0002 | -0.02 to 0.01 | 0.54 |

**Table S4. Antibacterial classification based on ATC code**

| **Antibacterial classification** | |
| --- | --- |
| **J01A Tetracyclines** | J01AA02 Doxycycline  J01AA08 Minocycline  J01AA12 Tigecycline |
| **J01C Beta-lactam antibacterials, penicillins** | J01CA01 Ampicillin  J01CA04 Amoxicillin  J01CE08 Benzathine benzylpenicillin  J01CF06 Nafcillin  J01CR01 Ampicillin/sulbactam  J01CR02 Amoxicillin/clavulanic acid  J01CR05 Piperacillin/tazobactam |
| **J01D Other beta-lactam antibacterials** | J01DB01 Cefalexin  J01DB04 Cefazolin  J01DB05 Cefadroxil  J01DB06 Cefazedone  J01DB11 Cefroxadine  J01DC01 Cefoxitin  J01DC02 Cefuroxime  J01DC04 Cefaclor  J01DC05 Cefotetan  J01DC07 Cefotiam  J01DC14 Flomoxef  J01DD01 Cefotaxime  J01DD02 Ceftazidime  J01DD04 Ceftriaxone  J01DD07 Ceftizoxime  J01DD08 Cefixime  J01DD13 Cefpodoxim  J01DD15 Cefdinir  J01DD16 Cefditoren  J01DD52 Ceftazidime/avibactam  J01DE01 Cefepime  J01DH02 Meropenem  J01DH03 Ertapenem  J01DH51 Imipenem  J01DI54 Ceftolozane/tazobactam |
| **J01E Sulfonamides and trimethoprim** | J01EE01 Trimethoprim/sulfamethoxazole |
| **J01F Macrolides, lincosamides and streptogramins** | J01FA06 Roxithromycin  J01FA09 Clarithromycin  J01FA10 Azithromycin  J01FF01 Clindamycin |
| **J01G Aminoglycoside antibacterials** | J01GB06 Amikacin  J01GB03 Gentamicin |
| **J01M Quinolone antibacterials** | J01MA02 Ciprofloxacin  J01MA12 Levofloxacin  J01MA14 Moxifloxacin |
| **J01X Other antibacterials** | J01XA01 Vancomycin (IV)  J01XA02 Teicoplanin  J01XB01 Colistin  J01XD01 Metronidazole (IV)  J01XE01 Nitrofurantoin  J01XX01 Fosfomycin  J01XX05 Methenamine  J01XX08 Linezolid  J01XX09 Daptomycin |
| **Other antibacterials** | A07AA09 Vancomycin (PO)  A07AA11 Rifaximin  B05CA05 Taurolidine  P01AB01 Metronidazole (PO) |

**Figure S1. Weekly trends in the use of antibiotic classes with segmented lines around ASP implementation**

**Figure S2. Antibiotic class-specific effects on *C. difficile* infection and colonization at fixed total DOT**

**(A) Antibiotic class-specific effects on *C. difficile* infection**

**(B) Antibiotic class-specific effects on *C. difficile* infection and colonization**
